# Antitrypanosomal activity of 5-nitro-2-aminothiazole-based compounds

**DOI:** 10.1016/j.ejmech.2016.04.010

**Published:** 2016-07-19

**Authors:** Maria V. Papadopoulou, William D. Bloomer, Howard S. Rosenzweig, Shane R. Wilkinson, Joanna Szular, Marcel Kaiser

**Affiliations:** a NorthShore University HealthSystem, Evanston, IL, USA; b Oakton Community College, Des Plaines, IL, USA; c School of Biological & Chemical Sciences, Queen Mary University of London, London, UK; d Swiss Tropical and Public Health Institute, Parasite Chemotherapy, Basel, Switzerland; e University of Basel, Basel, Switzerland

**Keywords:** 5-Nitro-2-aminothiazoles, Type I nitroreductase, Antitrypanosomal agents, Chagas disease, Leishmania, MZSRZQZBRGCXDO-UHFFFAOYSA-N, NTD, Neglected tropical diseases, *T. brucei*, *Trypanosoma brucei*, HAT, human African trypanosomiasis, *T. cruzi*, *Trypanosoma cruzi*, Bnz, benznidazole (N-benzyl-2-(2-nitro-1*H*-imidazol-1-yl)acetamide), Nfx, nifurtimox (4-(5-nitrofurfurylindenamino)-3-methylthio-morpholine-1,1-dioxide), NTR, type I nitroreductase, TbNTR, *T. brucei* NTR, CYP51, sterol 14α-demethylase enzyme, TcCYP51, *T. cruzi* CYP51, IC_50_, concentration for 50% growth inhibition, SI, selectivity index, SAR, structure-activity relationships

## Abstract

A small series of 5-nitro-2-aminothiazole-based amides containing arylpiperazine-, biphenyl- or aryloxyphenyl groups in their core were synthesized and evaluated as antitrypanosomatid agents. All tested compounds were active or moderately active against *Trypanosoma cruzi* amastigotes in infected L6 cells and *Trypanosoma brucei brucei*, four of eleven compounds were moderately active against *Leishmania donovani* axenic parasites while none were deemed active against *T. brucei rhodesiense*. For the most active/moderately active compounds a moderate selectivity against each parasite was observed. There was good correlation between lipophilicity (clogP value) and antileishmanial activity or toxicity against L6 cells. Similarly, good correlation existed between clogP values and IC_50_ values against *T. cruzi* in structurally related subgroups of compounds. Three compounds were more potent as antichagasic agents than benznidazole but were not activated by the type I nitrorectusase (NTR).

## Highlights

• Novel 5-nitro-2-aminothiazole-based amides were synthesized and evaluated for antiparasitic activity.
• Most derivatives were active or moderately active against *Trypanosoma cruzi* and *Trypanosoma brucei*.
• Some analogs demonstrated moderate antileishmanial activity *in vitro.*
• Lipophilicity positively affects antichagasic and antileishmanial activity.
• These compounds are not activated by Type I nitroreductases.

## Graphical abstract

**Figure:**
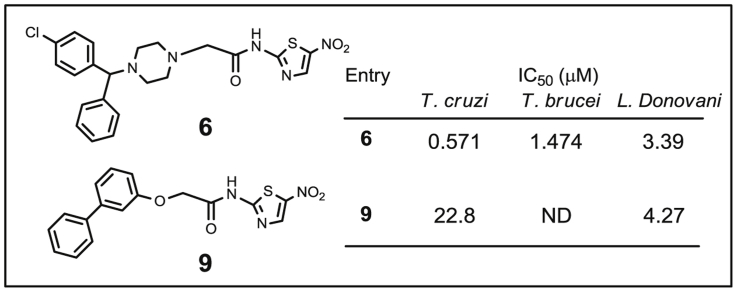

## 1 Introduction

American trypanosomiasis (Chagas disease), human African trypanosomiasis (HAT or sleeping sickness) and leishmaniasis are considered neglected tropical diseases (NTD) and represent a severe global health problem [1], [2]. It is estimated that together these three diseases, caused by protozoan parasitic infections, affect approximately 20 million people and are responsible for more than 110,000 deaths annually [2]. African trypanosomiasis is endemic in many sub-Saharan African countries and is caused by *Trypanosoma brucei rhodesiense* and *T. brucei gambiense*. Chagas disease, caused by *Trypanosoma cruzi*, is endemic in South and Central America but is now spreading worldwide, mainly due to human and vector migration [3], [4]. Leishmaniasis, caused by more than 20 *Leishmania* species, occurs throughout tropical and sub-tropical regions and is now spreading worldwide as an HIV co-infection [5].

Treatment of these NTD is currently based on a series of problematic drugs. Thus, nifurtimox (Nfx) and benznidazole (Bnz), the two currently used medications for Chagas disease are associated with limited efficacy, severe toxicity and long treatment requirements [6], [7]. Similarly, drugs used to treat HAT and leishmaniasis are highly toxic (e.g. melarsoprol, antimonials), may require i.v. administration (e.g. melarsoprol, suramin, DFMO, antimonials), can cause severe side effects, or are of high cost (e.g. DFMO, liposomal amphotericin B, miltefosine and paromomycin) [8], [9], [10]. Therefore, there is an urgent need for new effective, safe and affordable alternatives.

Although inhibitors of the fungal sterol 14α-demethylase enzyme (CYP51) and the orthologous enzyme *T. cruzi* CYP51 (TcCYP51) demonstrated promising efficacy against Chagas disease in preclinical studies [11], [12], [13], data from clinical trials using posaconazole or ravuconazole were disappointing [14], [15]. Moreover, recent evidence indicates that nitroheterocyclics might be more efficacious trypanocidal agents than CYP51 inhibitors [16], and combinations of the two may offer even a better solution [17].

We have shown that several chemical classes of 3-nitro-1*H*-1,2,4-triazole-based compounds exhibit excellent antichagasic activity both *in vitro* and *in vivo* [18], [19], [20], [21], [22], [23], [24], [25]. Furthermore, appreciable anti-HAT activity was also observed *in vitro* with several such analogs [18], [19], [20], [21], [22], [23], [24], [25] whereas *in vitro* antileishmanial activity was demonstrated with a sub-class of 3-nitrotriazole- and 2-nitroimidazole-based aryloxyphenylamides [25]. Nitro-activation by an oxygen-insensitive type I nitroreductase (NTR), an enzyme located in the mitochondrion of trypanosomatids and absent from most other eukaryotes, is partially responsible for the trypanocidal activity of these and other nitroheterocyclic compounds [18], [19], [21], [22], [23], [24], [25], [26], [27], [28], [29]. More recently, we have synthesized 3-nitrotriazole-based rigid amides and carbinols which act as bifunctional agents; they exert their antitrypanosomal activity upon activation by type I NTRs and by inhibiting the parasite's CYP51 enzyme [23], [25]. Interestingly, 3-nitrotriazole-based compounds are significantly more potent and less toxic than their 2-nitroimidazole-based counterparts [18], [19], [20], [21], [22], [23], [24], [25], [30].

Here we have expanded our research by investigating the role that another nitroheterocyclic ring, 5-nitro-2-aminothiazole, plays in antitrypanosomatid activity. Nitrothiazole- and nitrobenzothiazole-containing compounds exhibit antiparasitic, antibacterial, antifungal and antitubercular activities [31], [32], [33], [34]. Therefore, we have synthesized and evaluated *in vitro* a small series of 5-nitro-2-aminothiazole-based compounds bearing moieties that were previously proven effective in the trypanocidal activity of 3-nitrotriazole-based agents.

## 2 Results and discussion

### 2.1 Chemistry

**Scheme 1:**
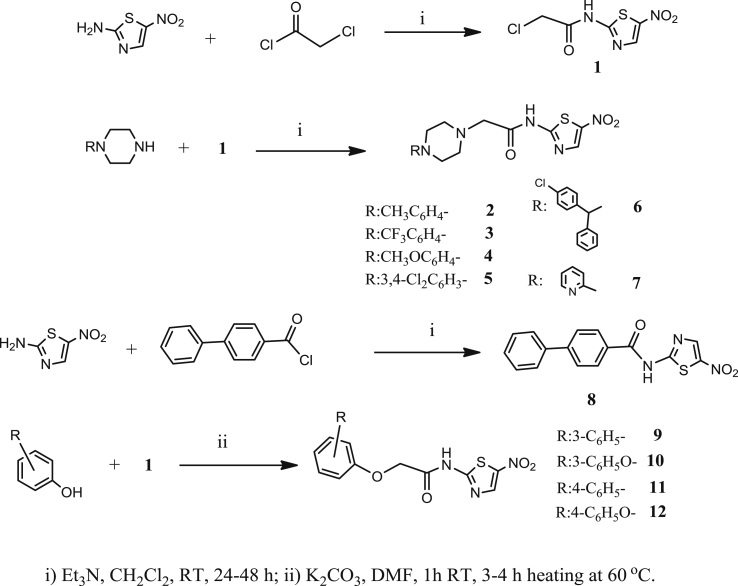
Synthesis of compounds on Table 1.

The synthesis of 5-nitro-2-aminothiazole-based compounds (Table 1) is straightforward and based on well-established chemistry, outlined in Scheme 1.

The precursor alkylchloride **1** as well as compound **8** were formed by nucleophilic substitution of 2-chloroacetyl chloride and [1,1′-biphenyl]-4-carbonyl chloride, respectively, by 5-nitro-2-aminothiazole, in the presence of triethylamine. Amides **2–7** were obtained by nucleophilic substitution of alkylchloride **1** by an appropriate piperazine at room temperature and in the presence of triethylamine. Finally, amides **9–12** were prepared by nucleophilic substitution of alkylchloride **1** by the potassium salt of an appropriate phenol in DMF, by heating for 3–4 h at 60 °C. Efforts were made to improve the yield of amides **9–12** by changing the solvent to anhydrous DMSO or CH_3_CN without any positive results. All final compounds and intermediates were characterized by ^1^H NMR (500 or 400 MHz) and HRMS.

### 2.2 Biological evaluation

#### 2.2.1 Antiparasitic activity and cytotoxicity

Compounds in Table 1 were screened for antiparasitic activity against three trypanosomatids: *T. cruzi*, *T. b. rhodesiense* and *Leishmania donovani*. The concentration of compound that inhibits parasite growth by 50% (IC_50_) was calculated from dose response curves for each parasite (Table 1). In addition, compounds were tested for toxicity in L6 rat skeletal myoblasts, used as host cells for *T. cruzi* amastigotes, in order to calculate a selectivity index for each parasite (SI = IC_50L6_/IC_50parasite_) (Table 1). Antiparasitic activity was evaluated according to the following criteria: an IC_50_ of <4.0 μM, between 4.0 and 60 μM or >60 μM, designates ‘active’, ‘moderately active’ or ‘inactive’ compounds, respectively, against *T. cruzi* amastigotes; for blood stream form (BSF) *T. b. rhodesiense*, IC_50_ values of <0.5 μM, between 0.5 and 6.0 μM or >6.0 μM identify ‘active’, ‘moderately active’ or ‘inactive’ compounds, respectively; finally, for *L. donovani* amastigotes, IC_50_ of <1 μM, between 1.0 and 6.0 μM or >6.0 μM, provides ‘active’, ‘moderately active’ or ‘inactive’ compounds, respectively [35].

According to the criteria set above, all tested compounds in Table 1 were active or moderately active antichagasic agents (green or light green, respectively). Four compounds (**6, 9, 10** and **12**) were moderately active (light green) against *L. donovani* parasites whereas no compound demonstrated antiparasitic activity against *T. b. rhodesiense.* Moreover, all compounds showed PSA values > 100 Å^2^, which makes them highly unlikely to be capable of penetrating the blood–brain barrier and demonstrate anti-HAT activity *in vivo.*

Several analogs (**3, 5–9**) demonstrated IC_50_ values < 50 μM against L6 host cells, presumably due to their high lipophilicity (Table 1), resulting in low selectivity indices. However, even compounds with IC_50_ values > 50 μM against L6 cells demonstrated a less than ideal SI, which is desired to be ≥ 50 for *T. cruzi* and ≥20 for *L. donovani* [35].

#### 2.2.2 SAR analysis of antichagasic activity

The compounds in Table 1 were synthesized having in mind 3-nitro-1,2,4-triazole-based analogs with known substantial trypanocidal properties, described previously by this group [20], [23], [24], [25]. Taking a closer look at the piperazine derivatives **2–7**, we observe that these yielded IC_50_ values against *T. cruzi* parasites ranging from 0.571 to 9.31 μM; thus they are 1.1- to 9-fold less potent than the corresponding 3-nitrotriazole-based analogs (IC_50_ values 0.169–4.64 μM) [24]. Similarly, the aryl/aryloxy-derivatives **8–12** were only moderately active antichagasic agents, compared to 3-nitrotriazole-based aryloxyphenylamides which demonstrate *T. cruzi* IC_50_ values at low nM concentrations [23], [25]. Therefore, clearly 5-nitro-2-aminothiazole-based amides are less potent antichagasic agents than their 3-nitrotriazole-based analogs.

Another general observation is that the 5-nitro-2-aminothiazole-based amides are significantly more lipophilic (Table 1) than their 3-nitrotriazole-based counterparts with the latter having clogP values between −0.198 and 3.1. In addition, 5-nitro-2-aminothiazole-based amides demonstrate higher PSA values than their 3-nitrotriazole-based analogs (the latter having PSA values less than 116 [24]), which may negatively affect cell permeation [36]. These features may contribute to the higher toxicity of the nitroaminothiazoles in L6 cells and their reduced potency against the parasites (Table 1).

**Fig. 1:**
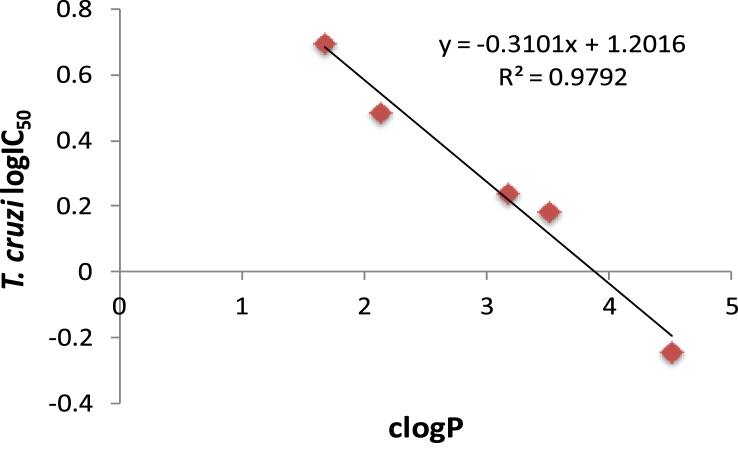
Correlation between antichagasic activity (log IC_50_ values against *T. cruzi*) and lipophilicity (clogP values) in **3**–**7**, compounds that are active against *T. cruzi* and bear a piperazine moiety.

There was an excellent correlation between antichagasic activity and lipophilicity (R^2^ = 0.979) in the piperazine-amide subgroup of compounds **3–7** (which were active against *T. cruzi*) as shown in Fig. 1. Thus, the piperazine amide **6** with the highest clogP value (4.51) was the most active compound against *T. cruzi*, demonstrating an IC_50_ of 571 nM, 3.86-fold more active than Bnz (Table 1). Compound **6**, however, was about 8-fold less active than its 3-nitrotriazole-based analog, in which the piperazinic ring is directly connected with the carbonyl (piperazide) and the nitrotriazole ring is connected with the carbonyl through a methylene group [24]. SAR follows the same rules observed in the 3-nitrotriazole-based piperazines and piperazides [20], [24]. Therefore, dichlorophenylpiperazine **5** was a slightly better antichagasic agent than trifluoromethylphenylpiperazine **3**, the latter demonstrating better antichagasic activity than the methoxyphenylpiperazine **4** or the heteroarylpiperazine **7**. It is not clear if the electronic effect of substitution plays a role in activity other than influencing the clogP value.

**Fig. 2:**
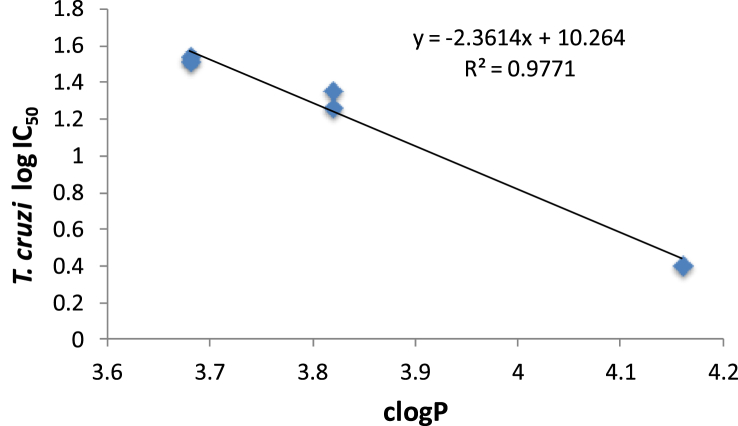
Correlation between antichagasic activity (log IC_50_ values against *T. cruzi*) and lipophilicity (clogP values) in the subgroup of structurally similar compounds **8–12**.

With regard to in the structurally related compounds **8–12**, there was also excellent correlation between antichagasic activity and lipophilicity (R^2^ = 0.977), with the most lipophilic biphenylamide **8** having the lowest IC_50_ value against *T. cruzi* (Fig. 2). Interestingly, and despite their relatively high lipophilicity, the (phenoxy/phenyl)phenoxy-derivatives **9–12** were only moderately active antichagasic agents, in stark contrast to 3-nitrotriazole-based aryloxyphenylamides, which were exceptionally active (at low nM concentrations) against *T. cruzi* [25].

#### 2.2.3 Analysis of anti-Leishmania activity

There was no correlation between antichagasic and antileishmanial activity for compounds **2–12**. Thus, compounds **9, 10** and **12** that displayed a moderate antichagasic activity (IC_50_ values of 23–35 μM) demonstrated high potency towards *L. donovani*, yielding IC_50_s of 4.27–5.40 μM.

**Fig. 3:**
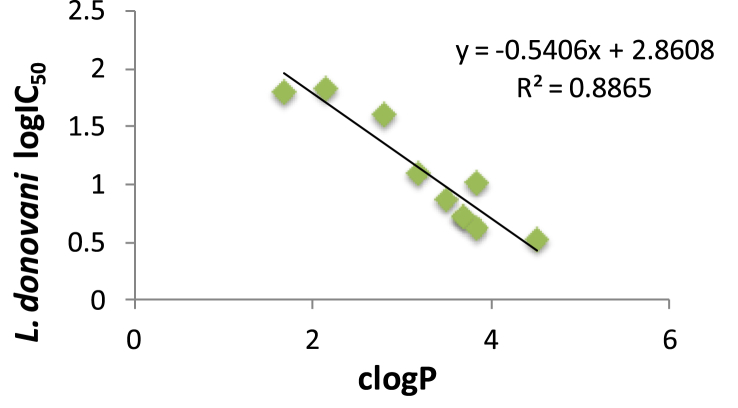
Correlation between log IC_50_ values against *L. donovani* and clogP values for compounds in Table 1.

**Fig. 4:**
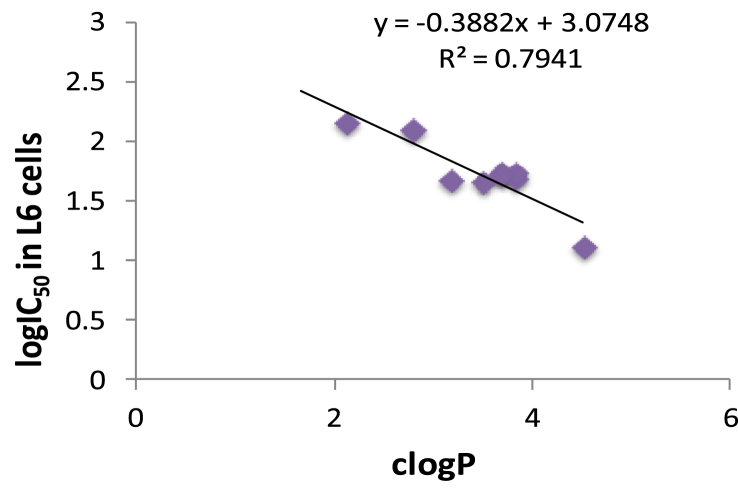
Correlation between log IC_50_ values in L6 cells and clogP values for compounds in Table 1.

In contrast to antichagasic activity and, with the exception of the most lipophilic compound **6**, the 3-nitro-2-aminothiazole-based piperazine amides **2–7** were generally less active antileishmanial agents than the (phenoxy/phenyl)phenoxy derivatives **9–12**. With regard to the role of substitution in the antileishmanial activity, the same rules existed, which were mentioned above for antichagasic activity. There was good correlation between clogP and logIC_50_ values against *L. donovani* parasites for all compounds (Table 1), regardless of activity (Fig. 3). Therefore, once again, lipophilicity was very important determinant for antileishmanial activity. However, lipophilicity also resulted in relatively high toxicity with good correlation between clogP values and logIC_50_ values in L6 cells (Fig. 4).

#### 2.2.4 The role of type I nitroreductases

**Fig. 5:**
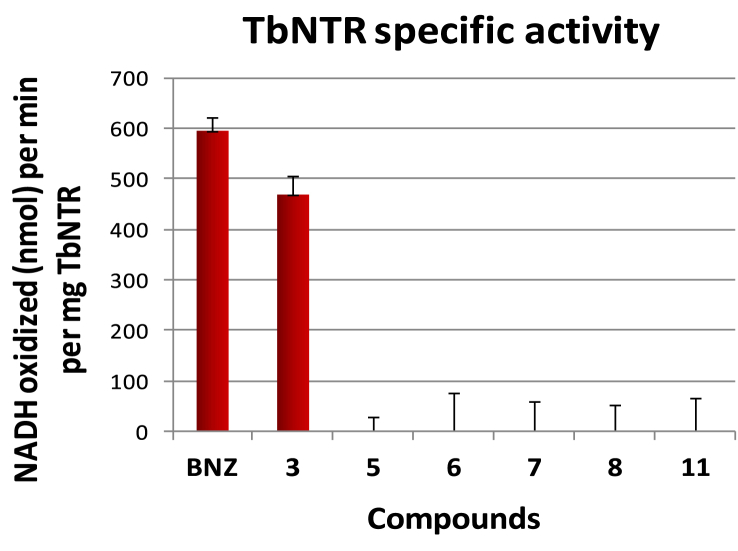
Specific activity values are measured in nmol NADH oxidized min^−1^ mg^−1^ TbNTR. The values correspond to averages from assays performed in triplicate ± standard deviation.

To elucidate the mechanism of action of the novel compounds in Table 1, representative derivatives (**3, 5, 6, 7, 8** and **11**) were evaluated as substrates of purified, recombinant TbNTR and their enzyme specific activity, measured as nmol NADH oxidized min^−1^ mg^−1^ protein, compared with that of benznidazole (Fig. 5). With the exception of compound **3**, all other tested analogs were deemed to be poor TbNTR substrates, consistent with results obtained for previously studied *N*-substituted 5-nitro-2-aminothiazoles where the substituent had electron withdrawing groups [37]. Compound **3**, which was not the most potent analog against *T. cruzi* amastigotes, provided a similar TbNTR specific activity to BNZ (Fig. 5).

To further determine whether NTR plays a role in metabolizing the substrates within the parasite, the above subset of compounds were phenotypically screened against BSF *T. b. brucei* expressing wild type or elevated levels of TbNTR. Compound **7**, with an IC_50_ > 10 μM in wild type parasites was not screened against the recombinant line. For most of the remaining compounds cells overexpressing TbNTR were only 2- to 3-fold more sensitivity than controls to the agent, a relatively low shift when compared to that observed with nifurtimox. The biochemical and phenotypic screening data suggest that NTR plays little or no role in the metabolism of these compounds within the parasite itself. As these ‘non-TbNTR’ activated compounds display moderate growth inhibitory properties towards wild type *T. b. brucei* (Table 2) the mode of action of these compounds remains unknown although this antitrypanosomal activity does appear to be sub-species specific: The selected 5-nitrothiazole-based compounds tested are up to 12-fold more potent towards *T. b. brucei* than against *T. b. rhodesiense*.

In conclusion, novel *N*-substituted 5-nitro-2-aminothiazoles with an arylpiperazine-, biphenyl- or aryloxyphenyl group in the core were active or moderately active antichagasic agents and moderately active against *T. b. brucei* parasites. Only one derivative, compound **6**, demonstrated activity against *T. cruzi* amastigotes at nM concentrations and was about 4-fold more potent than BNZ. In addition, some of these compounds demonstrated a moderate antileishmanial activity against *L. donovani* axenic amastigotes. These particular compounds were not good substrates for type I NTR. However, more simple *N*-substituted 5-nitro-2-aminothiazoles were shown to be excellent substrates of type I NTR and their antiparasitic activity was increased about 10-fold in NTR overexpressing *T. b. brucei* [37]. Interestingly, these more simple *N*-acyl substituted 5-nitro-2-aminothiazoles demonstrated significantly lower clogP values than the compounds described in here [37]. Therefore, this class of compounds deserves further investigation and structural optimization may provide leads for further development.

## 3 Experimental

### 3.1 Chemistry

#### 3.1.1 General

All starting materials and solvents were purchased from Sigma–Aldrich (Milwaukee, WI), were of research-grade quality and used without further purification. Solvents used were anhydrous and the reactions were carried out under a nitrogen atmosphere and exclusion of moisture. Melting points were determined by using a Mel-Temp II Laboratory Devices apparatus (Holliston, MA) and are uncorrected. Proton NMR spectra were obtained on a Varian Inova-500 or an Agilent Hg-400 spectrometer at 500 or 400 MHz, respectively, and are referenced to Me_4_Si or to the corresponding solvent, if the solvent was not CDCl_3_. High-resolution electrospray ionization (HRESIMS) mass spectra were obtained on a Agilent 6210 LC-TOF mass spectrometer at 11,000 resolution. Thin-layer chromatography was carried out on aluminum oxide N/UV_254_ or polygram silica gel G/UV_254_ coated plates (0.2 mm, Analtech, Newark, DE). Chromatography was carried out on preparative TLC alumina GF (1000 microns) or silica gel GF (1500 microns) plates (Analtech). All final compounds were purified by preparative TLC chromatography on silica gel or alumina plates and also checked by HPLC (≥95% purity).

#### 3.1.2 Synthesis of 2-chloro-N-(5-nitrothiazol-2-yl)acetamide (**1**) [33]

A suspension of 5-nitrothiazol-2-amine (1 eq) and triethylamine (1.1 eq) in 10 mL dichloromethane was added drop wise to a dichloromethane solution (5–7 mL) of 2-chloroacetyl chloride (1.1 eq) and the reaction was left overnight at room temperature. Alternatively, the solution of 2-chloroacetyl chloride was added at once to the suspension of 5-nitrothiazol-2-amine and triethylamine. The TLC on silica gel with ethyl acetate: petroleum ether (75:25) indicated completion of the reaction. The desired product was isolated as light yellow crystals through column chromatography as above: 481 mg (81% yield).

#### 3.1.3 General synthesis of N-(5-nitrothiazol-2-yl)acetamides **2–7**

A dichloromethane solution (6 mL) of an appropriate piperazine (1 eq) and triethylamine (3 eq) was added drop wise to a suspension of 2-chloro-N-(5-nitrothiazol-2-yl)acetamide (**1**) in 5 mL CH_2_Cl_2_ and the reaction was kept at room temperature under a nitrogen atmosphere and stirring for 48 h. The reaction solvent was evaporated and the residue was redissolved in ethyl acetate. The inorganic salts were filtered away and the residue was chromatographed on preparative TLC silica gel plates with ethyl acetate/petroleum ether as eluent to obtain the desired pure product as a powder or crystals. Purity was also checked by HPLC and it was ≥95%.

##### 3.1.3.1 N-(5-nitrothiazol-2-yl)-2-(4-(p-tolyl)piperazin-1-yl)acetamide (**2**)

Orange microcrystallic powder (62%): mp 160–161 °C (dec); ^1^H NMR (400 MHz, (CDCl_3_) δ: 8.33 (s, 1H), 7.10 (d, *J* = 7.6 Hz, 2H), 6.85 (d, *J* = 8.8 Hz, 2H), 3.38 (s, 2H), 3.23 (t, *J* = 4.8 Hz, 4H), 2.81 (t, *J* = 5.2 Hz, 4H), 2.29 (s, 3H). HRESIMS calcd for C_16_H_20_N_5_O_3_S *m/z* [M+H]^+^ 362.1281 found 362.1285.

##### 3.1.3.2 N-(5-nitrothiazol-2-yl)-2-(4-(4-(trifluoromethyl)phenyl)piperazin-1-yl)acetamide (**3**)

Orange powder (55%): mp 173–175 °C; ^1^H NMR (400 MHz, (CDCl_3_) δ: 8.33 (s, 1H), 7.51 (d, *J* = 8.4 Hz, 2H), 6.96 (d, *J* = 8.4 Hz, 2H), 3.40 (s, 2H), 3.38 (t, *J* = 5.2 Hz, 4H), 2.82 (t, *J* = 5.2 Hz, 4H). HRESIMS calcd for C_16_H_16_N_8_O_4_S *m/z* [M+H]^+^ 416.1010, found 416.1005.

##### 3.1.3.3 2-(4-(4-methoxyphenyl)piperazin-1-yl)-N-(5-nitrothiazol-2-yl)acetamide (**4**)

Bright orange powder (57%): mp 154–156 °C (dec); ^1^H NMR (400 MHz, (CDCl_3_) δ: 8.33 (s, 1H), 6.91 (d, *J* = 8.8 Hz, 2H), 6.86 (d, *J* = 9.2 Hz, 2H), 3.78 (s, 3H), 3.39 (s, 2H), 3.17 (t, *J* = 4.8 Hz, 4H), 2.81 (t, *J* = 4.8 Hz, 4H). HRESIMS calcd for C_16_H_20_N_5_O_4_S *m/z* [M+H]^+^ 378.1231 found 378.1233.

##### 3.1.3.4 2-(4-(3,4-dichlorophenyl)piperazin-1-yl)-N-(5-nitrothiazol-2-yl)acetamide (**5**)

Bright yellow microcrystals (65%): mp 169–171 °C; ^1^H NMR (400 MHz, (CD_3_COCD_3_) δ: 8.44 (s, 1H), 7.36 (d, *J* = 9.2 Hz, 1H), 7.12 (d, *J* = 2.8 Hz, 1H), 6.97 (dd, *J* = 9.2, 2.8 Hz, 1H), 3.53 (s, 2H), 3.37 (t, *J* = 5.2 Hz, 4H), 2.84 (t, *J* = 5.2 Hz, 4H). HRESIMS calcd for C_15_H_16_Cl_2_N_5_O_3_S *m/z* [M+H]^+^ 416.0345, 418.0317, found 416.0346, 418.0317.

##### 3.1.3.5 2-(4-((4-chlorophenyl)(phenyl)methyl)piperazin-1-yl)-N-(5-nitrothiazol-2-yl)acetamide (**6**)

Orange microcrystallic powder (66%): mp 92–95 °C; ^1^H NMR (400 MHz, (CDCl_3_) δ: 8.31 (s, 1H), 7.36–7.21 (m, 9H), 4.27 (s, 1H), 3.31 (s, 2H), 2.66 (t, *J* = 4.8 Hz, 4H), 2.49 (br s, 4H). HRESIMS calcd for C_22_H_23_ClN_5_O_3_S *m/z* [M+H]^+^ 472.1205, found 472.1209.

##### 3.1.3.6 N-(5-nitrothiazol-2-yl)-2-(4-(pyridin-2-yl)piperazin-1-yl)acetamide (**7**)

Orange microcrystallic powder (56%): mp 175–180 °C (dec); ^1^H NMR (400 MHz, (CDCl_3_) δ: 8.33 (s, 1H), 8.21 (dd, *J* = 4.4, 1.2 Hz, 1H), 7.52 (dt, *J* = 8.4, 2.0 Hz, 1H), 6.68 (m, 2H), 3.65 (t, *J* = 4.8 Hz, 4H) 3.38 (s, 2H), 2.76 (t, *J* = 4.8, Hz, 4H). HRESIMS calcd for C_14_H_17_N_6_O_3_S *m/z* [M+H]^+^ 349.1077 found 349.1083.

#### 3.1.4 N-(5-nitrothiazol-2-yl)-[1,1′-biphenyl]-4-carboxamide (**8**)

[1,1′-Biphenyl]-4-carbonyl chloride was added in portions to a suspension of 5-nitrothiazol-2-amine (1 eq) and triethylamine (2.5 eq) in 10–12 mL dichloromethane. The reaction mixture was kept at room temperature overnight under stirring and a nitrogen atmosphere. The desired product was obtained after preparative TLC on silica gel plates using ethyl acetate: petroleum ether (50:50) as eluent. Beige microcrystals (55%): mp > 230 °C; ^1^H NMR (400 MHz, (CD_3_COCD_3_) δ: 8.50 (s, 1H), 8.31 (d, *J* = 8.8 Hz, 2H), 7.92 (d, *J* = 8.8 Hz, 2H), 7.78 (d, *J* = 7.6 Hz, 2H), 7.53 (t, *J* = 7.6 Hz, 2H), 7.45 (t, *J* = 7.6 Hz, 1H). HRESIMS calcd for C_16_H_10_N_3_O_3_S *m/z* [M−H]^−^ 324.0448 found 324.0464.

#### 3.1.5 General synthesis of N-(5-nitrothiazol-2-yl)acetamides **9–12**

An appropriate phenol (1.05 eq) and K_2_CO_3_ (2.1 eq) were mixed together in dry DMF (4 mL) and stirred for an hour. Then a DMF solution (4 mL) of 2-chloro-*N*-(5-nitrothiazol-2-yl)acetamide (1 eq) was added through a funnel and the reaction mixture was heated at 60 °C for 3–4 h. The solvent was evaporated and the residue was chromatographed on silica gel preparative TLC plates using ethyl acetate: petroleum ether mixture as an eluent. The desired product is formed in relatively small yield (≤35%) and appears immediately after the unreacted phenol on TLC. Changing the solvent to CH_3_CN or DMSO did not improve the yield.

##### 3.1.5.1 2-([1,1′-biphenyl]-3-yloxy)-N-(5-nitrothiazol-2-yl)acetamide (**9**)

White microcrystals (25%): mp 148–150 °C; ^1^H NMR (400 MHz, (CClD_3_) δ: 9.94 (br s, 1H), 8.36 (s, 1H), 7.60–7.33 (m, 7H), 7.21 (dd, *J* = 2.4, 1.6 Hz, 1H), 6.96 (dd, *J* = 7.2, 2.4, Hz, 1H), 4.85 (s, 2H). HRESIMS calcd for C_17_H_14_N_3_O_4_S *m/z* [M+H]^+^ 356.0700, found 356.0696.

##### 3.1.5.2 N-(5-nitrothiazol-2-yl)-2-(3-phenoxyphenoxy)acetamide (**10**)

White microcrystals (34%, based on recovered phenol): mp 151–153 °C; ^1^H NMR (400 MHz, (CDCl_3_) δ: 9.84 (br s, 1H), 8.35 (s, 1H), 7.38 (t, *J* = 7.6 Hz, 2H), 7.30 (t, *J* = 8.0 Hz, 1H), 7.17 (t, *J* = 8.0 Hz, 1H), 7.05 (d, *J* = 7.6 Hz, 2H), 6.73 (dd, *J* = 8.0, 2.0 Hz, 1H), 6.68 (dd, *J* = 8.0, 2.4 Hz, 1H), 6.62 (t, *J* = 2.4 Hz, 1H), 4.74 (s, 2H). HRESIMS calcd for C_17_H_14_N_3_O_5_S *m/z* [M+H]^+^ 372.0648, found 372.0649.

##### 3.1.5.3 2-([1,1′-biphenyl]-4-yloxy)-N-(5-nitrothiazol-2-yl)acetamide (**11**)

Light yellow microcrystals (35%, based on recovered phenol): mp 196–198 °C; ^1^H NMR (400 MHz, CDCl_3_) δ: 8.36 (s, 1H), 7.60 (d, *J* = 8.8 Hz, 2H), 7.55 (dd, *J* = 8.0, 0.8 Hz, 2H), 7.44 (t, *J* = 7.2 Hz, 2H), 7.35 (t, *J* = 7.6 Hz, 1H), 7.06 (d, *J* = 8.8 Hz, 2H), 4.83 (s, 2H). HRESIMS calcd for C_17_H_14_N_3_O_4_S *m/z* [M+H]^+^ 356.070, found 356.0694.

##### 3.1.5.4 N-(5-nitrothiazol-2-yl)-2-(4-phenoxyphenoxy)acetamide (**12**)

Off white microcrystals (30%): mp 185–187 °C; ^1^H NMR (400 MHz, (CDCl_3_) δ: 9.91 (br s, 1H), 8.36 (s, 1H), 7.33 (t, *J* = 8.0 Hz, 2H), 7.10 (t, *J* = 7.6 Hz, 1H), 7.05–6.95 (m, 6H), 4.77 (s, 2H). HRESIMS calcd for C_17_H_14_N_3_O_5_S *m/z* [M+H]^+^ 372.0649, found 372.0655.

### 3.2 Biological evaluation

#### 3.2.1 In vitro screening

*In vitro* activity against *T. cruzi*, *T. b. rhodesiense, L. donovani* and cytotoxicity assessment using L6 cells (rat skeletal myoblasts) was determined using a 96-well plate format as previously described [38]. Data were analyzed with the graphic program Softmax Pro (Molecular Devices, Sunnyvale, CA, USA), which calculated IC_50_ values by linear regression from the sigmoidal dose inhibition curves.

#### 3.2.2 In vitro T. brucei brucei antiproliferating assays and susceptibility studies

*T. brucei brucei* bloodstream form parasites were seeded at 1 × 10^3^ mL^−1^ in 200 μL of growth medium containing different concentrations of a nitrothiazole or nifurtimox. Where appropriate, induction of the TbNTR was carried out by adding tetracycline (1 μg/mL). After incubation for 3 days at 37 °C, resazurin (2.5 μg per well) was added to each well and the plates incubated for a further 8 h. The cell density of each culture was determined as described before [26] and the IC_50_ established.

#### 3.2.3 Enzymatic activity studies with type I NTRs

Recombinant TbNTR was prepared and assayed as previously described [39], [40]. The activity of purified his-tagged TbNTR was assessed spectrophotometrically at 340 nm using various nitrothiazole substrates (100 μM) and NADH (100 μM) with the enzyme specific activity expressed as nmol NADH oxidized min^−1^ mg^−1^ of enzyme. Benznidazole was used as control substrate.

## Figures and Tables

**Table 1:**
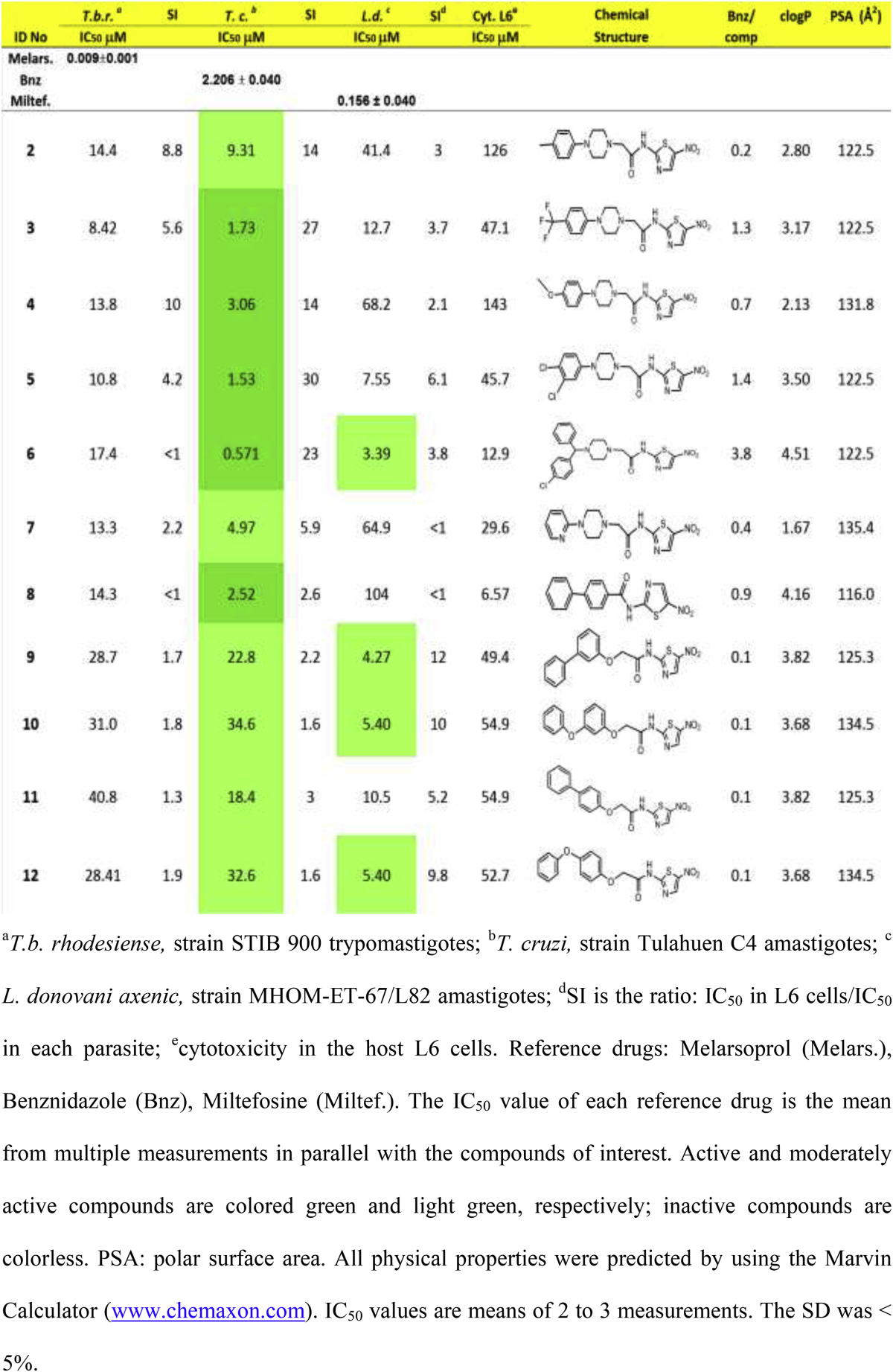
*In vitro* antiparasitic activity, host toxicity and physical properties of 5-nitro-2-amino-thiazole-based amides.

**Table 2:** Activity of selected nitrothiazoles against wild type and TbNTR overexpressing *T. b. brucei* parasites in the presence of tetracycline (+tet).

Compound	IC_50_ value (μM) *T.b. brucei*			Ratio
Wild type	TbNTR (−tet)	TbNTR (+tet)	–tet/+tet
nfx	3.980 ± 0.150	6.359 ± 0.119	0.869 ± 0.046	7.3
**3**	1.019 ± 0.045	1.460 ± 0.113	0.526 ± 0.024	2.8
**5**	1.365 ± 0.091	1.957 ± 0.101	0.747 ± 0.075	2.6
**6**	1.474 ± 0.104	1.638 ± 0.069	0.636 ± 0.022	2.6
**7**	21.575 ± 3.630	–	–	–
**8**	1.612 ± 0.396	4.587 ± 0.149	2.176 ± 0.085	2.1
**11**	3.753 ± 0.383	3.249 ± 0.142	2.200 ± 0.100	1.5

## Appendix A Supplementary data

### Mol Files

The following ZIP file contains the MOL files of the most important compounds referred to in this article.

MOL file. ZIP file containing the MOL files of the most important compounds in this article.
