# Supplementary material for: Antitrypanosomal activity of 5-nitro-2-aminothiazole-based compounds
Source: Eur J Med Chem. 2016 Jul 19;117:179–86. doi: 10.1016/j.ejmech.2016.04.010 (PMC4876673; doi:10.1016/j.ejmech.2016.04.010)
Supplement: Supplementary file 1 [file mmc1.docx]

**Antitrypanosomal activity of 5-nitro-****2-aminothiazole-based compounds**

Maria V. Papadopoulou^a^*, William D. Bloomer^a^, Howard S. Rosenzweig^b^, Shane R. Wilkinson^c^, Bhawana Gurung^c^, Joanna Szular^c^, Marcel Kaiser^d,e^

^a^*NorthShore University HealthSystem, Evanston, IL, US;* ^b^*Oakton Community College, Des Plaines, IL, US;* ^c^ *School of Biological & Chemical Sciences, Queen Mary University of London, London, UK;* ^d^*Swiss Tropical and Public Health Institute, Parasite Chemotherapy, Basel, Switzerland;* ^e^*University of Basel, Basel, Switzerland.*

**Representative ^1^H NMR spectra of compounds in Table 1.**

**Compound 2**

**Compound 4**

**Compound 9**

**Compound 10**

**Compound 12**

**Representative HRMS spectra of compounds in Table 1.**

**Compound 3**

**Compound 4**

**Compound 5**

**Compound 6**

**Compound 11**
